# 

**Published:** 1893-02

**Authors:** 


### CONTENTS—FEBRUARY, 1893—VOL. VIII., NO. 8.

ORIGINAL COMMUNICATIONS—

Recent cases of Cœliotomy. Dr. A. E. Spohn, Corpus Christi . . . 295

ABSTRACTS OF CURRENT MEDICAL LITERATURE—

Department of Therapeutics. Dr. David Cerna, Galveston . . . . . 310
Department of Practice of Medicine. Prof. A. J. Smith, Galveston . 314
Obstetrics and Gynecology. Prof. Wm. Kieller, Galveston . . . . 318
Department of Dermatology. Dr. Isadore Dyer, New Orleans . . . 324
Charity Hospital Clinical Reports. Robert Morris, New Orleans . . 327

EDITORIAL—

In Trouble . . . . . . . . . . . . . . . . . . . . . . . . . 331
The Texas Medical College . . . . . . . . . . . . . . . . . . 332
Death of Dr. Samuel Logan . . . . . . . . . . . . . . . . . . 333
Another Good Man Gone Wrong . . . . . . . . . . . . . . . . . 333

MEDICAL NEWS AND MISCELLANY . . . . . . . . . . . . . . . . . 334

PUBLISHERS' NOTES . . . . . . . . . . . . . . . . . . . . . . 337

